# The Fabrication of Cesium Lead Bromide-Coated Cellulose Nanocomposites and Their Effect on the Detection of Nitrogen Gas

**DOI:** 10.3390/s22207737

**Published:** 2022-10-12

**Authors:** Bumjun Park, Haneul Kang, Soobin Han, Hyeong-U Kim, Youngjin Cho, Yun Suk Huh, Sung-Min Kang

**Affiliations:** 1 Department of Biological Sciences and Bioengineering, Inha University, 100 Inha-ro, Michuhol-gu, Incheon 22212, Korea; 2 Department of Plasma Engineering, Korea Institute of Machinery & Materials, Daejeon 34103, Korea; 3 Food Safety and Distribution Research Group, Korea Food Research Institute, 245 Nongsaengmyeong-ro, Iseo-myeon, Wanju-gun 55365, Jeollabuk-do, Korea; 4 Department of Green Chemical Engineering, Sangmyung University, 31 Sangmyungdae-gil, Cheonan 31066, Chungcheongnam-do, Korea; 5 Future Environment and Energy Research Institute, Sangmyung University, 31 Sangmyungdae-gil, Cheonan 31066, Chungcheongnam-do, Korea

**Keywords:** cesium lead bromide nanofibers, cesium lead bromide nanocrystals, cellulose nanofibers, nitrogen gas, hot injection method, electrospinning

## Abstract

In this work, we fabricate cesium lead bromide nanofibers (CsPbBr_3_ NFs) via the attachment of cesium lead bromide nanocrystals (CsPbBr_3_ NCs) on the surface of electrospun cellulose nanofibers (CNFs) and employ them in a sensor to effectively detect gaseous nitrogen. The CsPbBr_3_ NFs are produced initially by producing CsPbBr_3_ NCs through hot injection and dispersing on hexane, followed by dipping CNFs and ultrasonicate for 1 h. Morphological characterization through visual, SEM and TEM image, and crystalline structure analysis by XRD and FT-IR analysis of CsPbBr_3_ NFs and NCs show similar spectra except for PL due to unavoidable damage during the ultrasonication. Gaseous nitrogen is subsequently detected using the photoluminescence (PL) property of CsPbBr_3_ NFs, in which the PL intensity dramatically decreases under various flow rate. Therefore, we believe that the proposed CsPbBr_3_ NFs show significant promise for use in detection sensors in various industrial field and decrease the potential of fatal damage to workers due to suffocation.

## 1. Introduction

Nitrogen is a colorless and odorless inert gas accounting for 78% of the atmospheric air that causes little chemical reaction [1]. Due to the properties of nitrogen, it is used in various industrial fields to prevent oxidation or to prevent accidents such as fire [2]. Moreover, nitrogen is an essential element of fertilizer required for plant growth [3]. However, the colorless, odorless, and depression characteristics of nitrogen gas can be rather toxic in industrial sites, in which nitrogen is constantly used to prevent corrosion of products. When the gas is leaked, it is difficult to immediately identify, followed by fatal damage to workers due to suffocation as the concentration of oxygen in the atmosphere decreases [4,5]. Therefore, it is necessary to develop a sensor that can efficiently detect nitrogen gas in industrial fields.

Nitrogen gas is detected through sensors that measure oxygen concentration or nitrogen oxide in preparation for nitrogen leakage in most industrial sites [6,7]. The fabricated sensors are operated as the leaked nitrogen lowers the oxygen or nitrogen oxide concentration, making it difficult to determine the immediate leakage of nitrogen [8]. Therefore, optical sensors can replace current sensors, which has the advantage of not only possibly responding to the leakage of nitrogen gas faster than existing sensors, but also directly detect the nitrogen gas, not through the detection of alternatives. Currently known optical sensors include chlorophyll meters, canopy reflectance sensors, and fluorescence-based flavonols meters, which are mostly used to determine the nitrogen state of the vegetable crop [9,10,11]. For example, chlorophyll meters measure the chlorophyll in the surface area, which is sensitive to nitrogen, and thus, nitrogen contained in leaf can be measured by transmission or fluorescence [12]. However, using a nitrogen-detectable optical sensor for existing vegetable crop is not suitable in an industrial field.

Perovskite light-emitting materials, have strong absorption characteristics, bright fluorescence, narrow full width at half maximum (FWHM) value, and high charge mobility and defect tolerance, leading to various applications such as solar cells, photodetectors, light-emitting diodes (LED), and sensors [13,14,15,16]. Among them, all inorganic cesium lead halide perovskite nanocrystals (CsPbX_3_ NCs, X=Cl, Br or I), compared with organic-inorganic hybrid perovskites, have superior characteristics on stability and narrow color purity fluorescence over a wide band of absorbance [17]. Therefore, application studies on various optoelectronic materials have been actively conducted using these excellent optical properties. For example, Maity et al. successfully fabricated CsPbX_3_ NCs-coated film that showed specific recognition behavior towards ammonia gas at room temperature [18]. Therefore, it is determined that CsPbX_3_ NCs with remarkable characteristics could be applied as an excellent sensor that detect nitrogen gas.

In this regard, photoluminescence sensors based on CsPbBr_3_ NCs attached on cellulose nanofibers (CNFs), so-called cesium lead bromide nanofibers (CsPbBr_3_ NFs), and detection availability on nitrogen gas are further determined. CsPbBr_3_ NCs are synthesized through a hot injection method in which two different precursors are quickly reacted under high temperature, leading to the nucleation of nanocrystals, while CNFs are fabricated by electrospinning technology, in which nanofibers are uniformly and abundantly formed as a mat when high voltage is applied on cellulose solution. Subsequently, CsPbBr_3_ NCs are successfully attached on the surface of CNFs through ultrasonication, CsPbBr_3_ NFs are finally fabricated. Generated CsPbBr_3_ NFs are then placed on various concentration of nitrogen gas, and the decrease of luminescence is observed. Therefore, CsPbBr_3_ NFs-based optical sensors are believed as next generation sensors in industrial field to detect exposed nitrogen gas effectively and directly.

## 2. Materials and Methods

### 2.1. Materials

Cellulose acetate (C_164_H_174_O_111_, average M_n_~30,000), cesium carbonate (Cs_2_CO_3_, 99%), lead(II) bromide (PbBr_2_, 99.999% trace metal basis), 1-octadecene (ODE; C_18_H_36_, technical grade 90%), oleylamine (OLA; C_18_H_37_N, technical grade 70%) and hexane (C_6_H_14_) were purchased from Sigma-Aldrich Chemicals (St. Louis, MO, USA). N,N-dimethylacetamide (DMA; C_4_H_9_NO), acetone (C_3_H_6_O, 99.5%) and oleic acid (OA; C_18_H_34_O_2_) were obtained from Daejung Chemicals & Metals Co., Ltd. (Siheung, Korea).

### 2.2. Synthesis of Cesium Lead Bromide Nanocrystals through Hot-Injection Method

Cesium lead bromide nanocrystals (CsPbBr_3_ NCs) were produced through hot-injection method. Two precursors, which were cesium oleate (Cs-oleate) and lead bromide precursors (PbBr_2_ precursors) were prepared. Cs-oleate was initially prepared by mixing 0.352 g of Cs_2_CO_3_ and 10.0 mL of ODE, followed by reacting at 120.0 °C for 1 h under nitrogen atmosphere. After the reaction, 0.65 mL of OA was added and the rection was continued for 30 min to form Cs-oleate. PbBr_2_ precursors were prepared by adding 0.356 g of PbBr_2_ and 20.0 mL of ODE, followed by reacting at 120.0 °C for 1 h under nitrogen atmosphere. Next, 4 mL of OA and OLA were added and kept on a reaction in a same condition for 15 min. Subsequently, the temperature of PbBr_2_ precursors was raised to 200.0 °C for 5 min, followed by rapid addition of 3.2 mL of Cs-oleate in precursors, the nucleation of the solutions occurred and CsPbBr_3_ NCs were successfully fabricated. Formed CsPbBr_3_ NCs were centrifuged at 10,000 rpm for 10 min, followed by discard of supernatant fluid and addition of 20 mL of hexane.

### 2.3. Fabrication of Cesium Lead Bromide Nanofibers

Initially, cellulose nanofibers (CNFs) were produced through electrospinning. The solution of 20 wt.% cellulose solution was prepared by mixing 2.0 g of cellulose acetate on 10.0 mL of mixed acetone and DMA with the ratio of 2:1. The prepared solution was then electrospun through a high-voltage supply (Nano NC, Seoul, Korea) and a syringe pump (Legato 100, KD Scientific, Holliston, MA, USA) under an applied voltage of 16.0 kV and 21-gauge single use injection needles with a flow rate of 1.0 mL h^−1^.

Cesium lead bromide nanofibers (CsPbBr_3_ NFs) were further fabricated through coating the surface of CNFs with CsPbBr_3_ NCs. CsPbBr_3_ NCs were uniformly coated on the surface of CNFs by ultrasonication bath. First, CNFs were well dipped on CsPbBr_3_ NCs-dispersed hexane and ultrasonicated for 1 h. Subsequently, well-coated CNFs were dried in a vacuum oven in set as 70 °C overnight, which finally formed CsPbBr_3_ NFs.

### 2.4. Detection of Nitrogen Gas through Fabricated Cesium Lead Bromide Nanofibers

The detection of nitrogen gas was carried out inside the gas sensor device. Initially, fabricated CsPbBr_3_ NFs were cut into 15 × 15 mm sample and placed on the glass of the same size. UV light was emitted through sapphire glass in the sensor device using external light source. The photoluminescence (PL) signals of the CsPbBr_3_ NFs were measured through a detector and a spectrophotometer connected to the computer device. Pure nitrogen gas (99.9%), the target gas, and air composed of 78% of nitrogen and 21% of oxygen, the carrier gas, was then injected to CsPbBr_3_ NFs at a flow rate of 100.0 sccm using a mass flow controller (MFC; GMC1200, ATOVAC, Yongin, Korea). The PL spectra of CsPbBr_3_ NFs were recorded 0, 5, 10, 15, 20, 25 and 30 min after the injection of nitrogen gas.

### 2.5. Characterization

Morphological characterization of the synthesized CsPbBr_3_ NFs, NCs and CNFs were conducted using high-resolution scanning electron microscopy (HR-SEM; SU8010, Hitachi, Japan) and field-emission transmission scanning electron microscopy (FE-TEM; JEM-2100F, JEOL, Tokyo, Japan). The crystalline structure was further analyzed using high-resolution X-ray diffraction (HR-XRD; X’Pert-PRO MRD, Philips, Amsterdam, The Netherlands) and Fourier-transform infrared spectroscopy (FT-IR; JASCO FT-IR 6600, Tokyo, Japan) in the range of 400 to 4000 cm^−1^. The PL intensity of the samples were recorded using Raman spectroscopy (RAON-Spec, NOST, Seongnam, Korea). Chemical composition and state of the samples were conducted using X-ray photoelectron spectroscopy (XPS; Thermo Fisher Scientific K-alpha System, Waltham, MA, USA). Finally, the size of the CsPbBr_3_ NFs and NCs were estimated using ImageJ software.

## 3. Results

### 3.1. Formation of Cesium Lead Bromide Nanocrystals-Coated Cellulose Nanofibers

**Figure 1:**
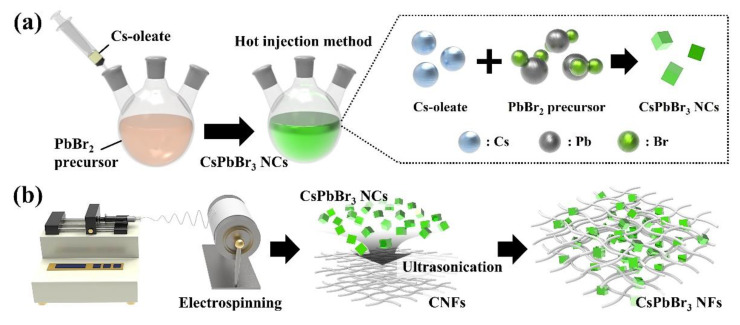
Schematic illustration of CsPbBr_3_ NFs fabrication; (**a**) synthesis of CsPbBr_3_ NCs through hot injection method, and (**b**) fabrication of CNFs by electrospinning, followed by production of CsPbBr_3_ NFs with attachment of CsPbBr_3_ NCs on the surface of CNFs.

CsPbBr_3_ NFs were fabricated by the integration of CsPbBr_3_ NCs and CNFs, which were synthesized through hot injection and electrospinning, respectively, as shown in Figure 1. Initially, hot injection was the way to synthesize CsPbBr_3_ NCs at high temperature using ionic chemical bonds in three-component compounds that were difficult to dissolve in a normal solvent (Figure 1a) [19]. CsPbBr_3_ NCs could be produced by reacting two precursors, which were Cs-oleate and PbBr_2_ precursors, under high temperature of 200.0 °C. At this time, the precipitation of Cs^+^, Pb^2+^ and Br^−^ ions might be controlled by the reaction in the high boiling point solvent ODE, and NCs were stabilized in the colloidal phase using OA and OLA mixture [20]. Therefore, it is possible to evenly produce CsPbBr_3_ NCs with a size of 4 to 15 nm in large quantities when NCs were synthesized using the hot injection method compared with the existing synthesis method.

CNFs were generated through electrospinning having a uniform size to a constant thickness (Figure 1b). Electrospinning, which could make a polymer in a form of nanofibers, used an external strong electric field to radiate fibers in a non-contact manner, thus obtaining fibers having a much thinner and uniform diameter [21]. In the case of CNFs, the charge was uniformly distributed on the surface of the droplets of the cellulose solution supplied from the needle as an electric field was applied, and a conical droplet called Taylor cone was formed and discharged toward the collector when the electrostatic repulsive force exceeded the surface tension [22]. As a result, randomly electrospun CNFs were obtained as a nonwoven membrane on the surface of the collector. CNFs produced in this way were supported on CsPbBr_3_ NCs dispersed in hexane, and NCs were adhered to the surface of CNFs after ultrasonication, resulted in the formation of CsPbBr_3_ NFs.

### 3.2. Characterization of Cesium Lead Bromide Nanocrystals-Coated Cellulose Nanofibers

**Figure 2:**
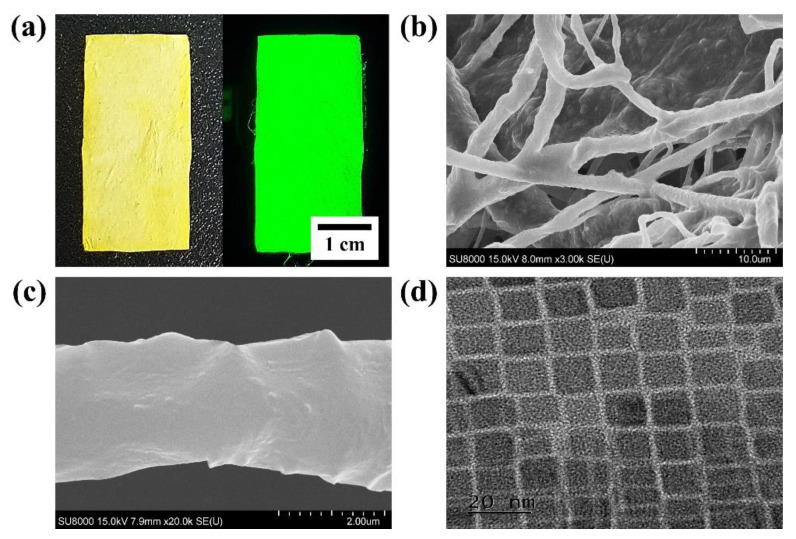
Morphological characterization of CsPbBr_3_ NFs and NCs; (**a**) CsPbBr_3_ NFs under visible light and UV field, scanning electron microscopy (SEM) images at (**b**) low and (**c**) high magnification, and (**d**) transmission electron microscopy (TEM) image of NCs.

Morphological characterization of fabricated CsPbBr_3_ NFs was specifically analyzed through visual, scanning electron microscope (SEM) and transmission electron microscope (TEM) as shown in Figure 2. First, CsPbBr_3_ NFs were generally yellowish under bright field, while the green fluorescence was confirmed under UV field (Figure 2a). This indicated that there was no significant difference from the fluorescence of CsPbBr_3_ NCs, in which the fluorescence did not change even though NCs were attached to CNFs through ultrasonication (Figure S1a). In addition, SEM image analysis confirmed that the general size of CsPbBr_3_ NFs was slightly deformed as NCs were coated on the surface of CNFs with a uniform size (Figure 2b). The surface of the CNFs was roughly changed due to the adhesion of CsPbBr_3_ NCs when analyzed under high magnification (Figure 2c). The results showed a significant difference from the SEM images of pure CNFs, which had a smooth surface before the attachment of NCs and verified that the surface change was due to the adhesion of NCs (Figure S1b). Finally, TEM images confirmed that CsPbBr_3_ NCs were successfully and regularly attached to the surface of CNFs (Figure S1c). The size of CsPbBr_3_ NCs synthesized through hot injection was approximately 6.92 ± 1.51 nm, while the size of NCs attached on the surface of NFs was 6.81 ± 1.40 nm, showing no big difference (Figure S2). Moreover, CsPbBr_3_ NCs were well distributed on the surface of CNFs without forming agglomerates when compared to pure NCs (Figure 2d and Figure S1d).

**Figure 3:**
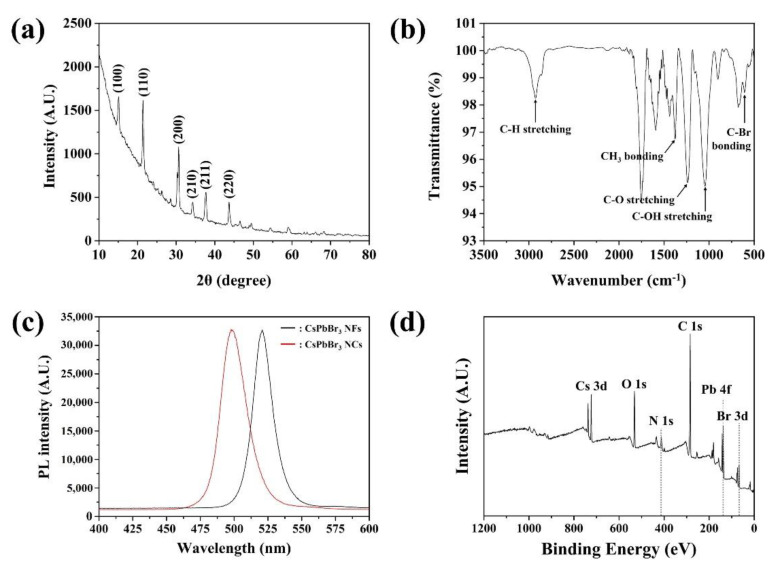
(**a**) X-ray diffraction (XRD) pattern, (**b**) Fourier-transform infrared (FT-IR) spectra, (**c**) photoluminescence (PL) spectra, and (**d**) wide scan X-ray photoelectron spectroscopy (XPS) analysis of the fabricated CsPbBr_3_ NFs.

The XRD diffractograms of CsPbBr_3_ NFs were analyzed to verify their crystalline structures (Figure 3a). CsPbBr_3_ NFs showed 2θ values of 15.14°, 21.53°, 30.73°, 34.37°, 37.74° and 43.81°, corresponding to the (100), (110), (200), (210), (211) and (220) planes, respectively, indicating the cubic crystalline structure of Pm−3m space group [23,24]. The curved baseline was formed due to CNFs backbone [25]. Interestingly, the characteristic peaks were equal to CsPbBr_3_ NCs synthesized through hot injection method, indicating that crystalline structure of attached NCs were not affected by ultrasonication during fabrication process of CsPbBr_3_ NFs (Figure S3). The Fourier transform infrared (FT-IR) spectra of CsPbBr_3_ NFs were further recorded to investigate the different chemical and functional groups (Figure 3b). The results showed that common peaks appeared at 2962.62, 1376.71, 1234.64 and 1043.94 cm^−1^, which were attributed to C−H stretching, CH_3_ bonding, C−O stretching, and C−OH bonding, respectively [26]. The appeared peaks all contributed to the molecular structure of CNFs. The characteristic peak was attributed at 674.06 cm^−1^ associated to C-Br bonding, which verified that CsPbBr_3_ NCs were well attached on carbon molecule placed on the surface of CNFs [27]. Moreover, fabricated CsPbBr_3_ NFs and CsPbBr_3_ NCs presented the emission spectra as depicted in Figure 3c. Both NFs and NCs displayed Gauss curves with sharp emission peaks position centered at 520.82 and 489.46 nm with narrow FWHM value of 16.12 and 21.16 nm, respectively. As NCs were attached on the surface of CNFs, the emission peak had red shifted due to unavoidable damage during ultrasonication.

The chemical composition and valence state of CsPbBr_3_ NFs were examined by X-ray photoelectron spectroscopy (XPS). The full-survey XPS spectrum of CsPbBr_3_ NFs in Figure 3d confirmed the coexistence of C, O, N, Cs, Pb and Br with atomic ratios of 77.33%, 13.81%, 1.88%, 1.43%, 1.08% and 4.47%, respectively. For further evaluation, deconvolution of the XPS spectra was performed (Figure S4, Table S1). The high-resolution XPS spectrum for C 1s displayed three peaks with binding energies of 283.93, 285.45, and 288.22 eV, corresponding to the C−C, C−O and C=O transitions exhibited in CNFs, respectively (Figure S4a) [28]. In case of O 1s XPS spectrum shown in Figure S4b, two peaks with binding energies of 531.12 and 532.22 eV were observed, which could be ascribed to C=O and C−O transitions, respectively, which also existed in CNFs [29]. The peaks of N 1s spectra of CsPbBr_3_ NFs were shown at 398.78 and 400.61 eV, which corresponded to metal nitride and C−NH_2_ respectively (Figure S4c) [30]. The Cs 3d peaks were deconvoluted into two characteristic peaks corresponding to Cs 3d_5/2_ and Cs 3d_3/2_ peaks at 723.43 eV and 737.41 eV (Figure S4d) [31]. Moreover, Pb 4f peaks displayed two characteristic peaks with binding energies of 137.22 and 142.06 eV, which corresponded to Pb 4f_7/2_ and Pb 4f_5/2_ (Figure S4e) [32]. Finally, three characteristic peaks were observed in the Br 3d spectrum, in which the binding energies of Br 3d_5/2_, Br 3d_3/2_ and C−Br were 67.19, 68.10 and 74.24 eV, respectively (Figure S4f) [33]. The presence of Cs, Pb and Br all indicated that CsPbBr_3_ NCs were well presented on the surface of CNFs. In addition, the presence of C-Br verified that the chemical bond between NCs and CNFs occurred between C and Br, which matched well with FT-IR analysis. Therefore, the XPS results strongly confirmed that CsPbBr_3_ NFs were well synthesized as CsPbBr_3_ NCs were formed and attached to the surface of CNFs.

### 3.3. Detection of Nitrogen Gas through Ceisum Lead Bromide-Coated Cellulose Nanofibers

**Figure 4:**
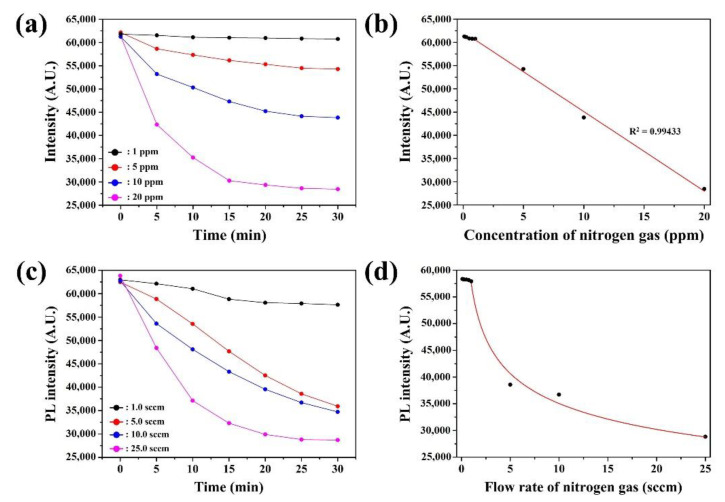
(**a**) Response of CsPbBr_3_ NFs at 520 nm under varios nitrogen gas concentration from 1 to 20 ppm and (**b**) sensitivity of CsPbBr_3_ NFs-based nitrogen gas sensor in concentration range of 0.1 to 20 ppm after 30 min of exposure time. (**c**) Response of CsPbBr_3_ NFs at 520 nm with various nirogen gas flow rate from 1.0 to 25.0 sccm, and (**d**) sensitivity of CsPbBr_3_ NFs-based nitrogen gas sensor in flow rate from 1.0 to 25.0 sccm after 25 min of exposure time.

Nitrogen gas detection was conducted using the proposed CsPbBr_3_ NFs under various concentrations. Our proposed sensing platform can achieve the rapid diagnosis of nitrogen gas with a high level of sensitivity and accuracy. To investigate the sensing performance of CsPbBr_3_ NFs, we carried out experiment for nitrogen gas detection under various concentrations (Figure 4). In general, lead halide-based perovskites are shown the atmosphere-dependent degradation mechanisms including lattice shrinkage and phase segregation, which can be induced to decrease the stability of the crystal structure, resulting the PL quenching [34,35]. First, the detection of nitrogen gas was tested by exposing the CsPbBr_3_ NFs to different concentrations (1 to 20 ppm) at room temperature (Figure 4a). It was observed that PL intensity decreased under all concentrations of nitrogen gas. It was also shown that the PL intensity of CsPbBr_3_ NFs was sensitive to concentrations below 5 ppm. The PL intensity at 30 min was further represented in Figure 4b with various concentration of nitrogen gas. It was observed that the PL intensity was similar at 30 min with the concentration between 0.1 to 1 ppm of nitrogen gas. However, the change in the PL intensity with 1 to 20 ppm of nitrogen gas was proportional to concentration with R2 value of 0.99433. The concentration of nitrogen gas was distinguishable by the measured PL intensity over 1 ppm.

**Figure 5:**
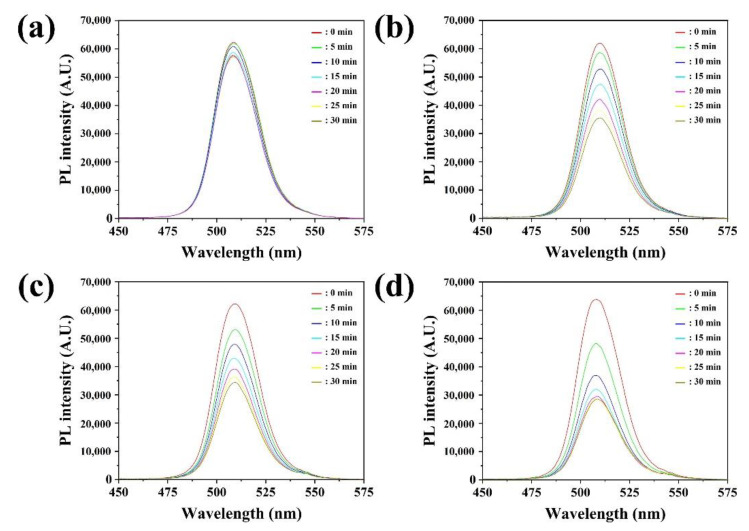
Normalized PL spectra of CsPbBr_3_ NFs exposed to nitrogen gas with flow rate of (**a**) 1.0 sccm, (**b**) 5.0 sccm, (**c**) 10.0 sccm and (**d**) 25.0 sccm.

The detection of nitrogen gas was further tested by exposing CsPbBr_3_ NFs to different flow rate from 1.0 sccm to 25.0 sccm in N_2_ carrier gas at room temperature (Figure 4a). The changes in the PL intensity of the CsPbBr_3_ NFs with exposure time were presented in Figure 5. The PL intensity was rapidly reduced within 30 min when the injected flow rate was high. However, the PL intensity was slowly decreased when the flow rate was low as 1.0 sccm. The similar response at low nitrogen gas flow rate was due to the influence of humidity, in which the contact between water molecules and CsPbBr_3_ NCs on the surface of CNFs degraded the PL properties of NCs. When water molecules settled on the surface of hydrophilic surface of CNFs matrix, nitrogen gas was trapped on the surface, leading the reduction of PL of CsPbBr_3_ NCs [36]. The response of gas sensor reached to almost maximum value after 25 min of nitrogen gas injection. The response at 25 min was represented in Figure 4b with various flow rate of nitrogen gas. It was observed that the decrease of PL intensity was well fit in curve at 25 min with the flow rate between 1.0 to 25.0 sccm of nitrogen gas. Therefore, the results suggested that detection of nitrogen gas under gaseous state through CsPbBr_3_ NFs had individual practical potential for on-site detection in industrial field.

The PL intensity in the presence of various gas was further observed to determine the selectivity of fabricated gas sensors (Figure S5a). Initially, the PL intensity of CsPbBr_3_ NFs dramatically decreased in the nitrogen gas. In comparison, with the addition of gas such as argon and oxygen, a change in the PL intensity did not occur. This indicates that the PL intensity is not strongly affected when different gases come into contact with the CsPbBr_3_ NFs. Additionally, the repeatability of fabricated gas sensors under nitrogen gas was measured (Figure S5b). Interestingly, the recovery did not occur when the sensors were exposed to the atmosphere right after the detection of nitrogen gas. The results indicated that the fabricated CsPbBr_3_ NFs-based sensors were one-time usable nitrogen gas detectable sensors.

## 4. Conclusions

In summary, we successfully fabricated CsPbBr_3_ NFs through the attachment of uniformly synthesized CsPbBr_3_ NCs onto the surface of electrospun CNFs and developed as a gas sensor aimed at detecting nitrogen gas. The fabricated CsPbBr_3_ NFs exhibited green fluorescence after attachment of NCs on the surface of CNFs, in which the structure of the NCs did not collapse during ultrasonication process. Further, the crystalline structure of the CsPbBr_3_ NCs stuck on the surface of CNFs was not disrupted and maintained as cubic structure, which was confirmed through XRD analysis. In addition, C-Br bonding clearly occurred, indicating that the Br atom on CsPbBr_3_ NCs bonded to the C atom on CNFs. The proposed CsPbBr_3_ NFs also demonstrated high sensitivity of 1 ppm to nitrogen under gaseous state as the PL intensity was strongly decreased. This was comparable with other detecting sensors as shown in Table S2, in which most previous research focused on various nitrogen-included gases such as ammonia, nitrogen dioxide and nitrogen oxide, while our research focused on pure nitrogen gas. Consequently, we believe that the developed sensor will be useful to detect nitrogen gas in industrial field compared to the conventional sensor.

## Data Availability

Not applicable.

## Supplementary Materials

The following supporting information can be downloaded at: https://www.mdpi.com/article/10.3390/s22207737/s1, Figure S1: Morphological characterization of CsPbBr_3_ NCs (a) under visible light and UV field, (b) SEM image of CNFs, TEM image of NCs under (c) low and (d) high magnification, Figure S2: Size distribution graph of (a) CsPbBr_3_ NFs and (b) NCs, Figure S3: X-ray diffraction (XRD) pattern of fabricated CsPbBr_3_ NCs through hot injection method, Figure S4: Narrow X-ray photoelectron spectroscopic (XPS) scan of (a) C 1s, (b) O 1s, (c) N 1s, (d) Cs 3d, (e) Pb 4f and (f) Br 3d for CsPbBr_3_ NFs, Figure S5: (a) Selectivity and (b) reusability of the fabricated gas sensor. Table S1. Deconvolution of the XPS spectra. Table S2. Comparison of gas sensors for detection of various types of nitrogen gas [37,38,39].
