# Supplementary material for: The Fabrication of Cesium Lead Bromide-Coated Cellulose Nanocomposites and Their Effect on the Detection of Nitrogen Gas
Source: Sensors (Basel). 2022 Oct 12;22(20):7737. doi: 10.3390/s22207737 (PMC9611072; doi:10.3390/s22207737)
Supplement: Supplementary file 1 [file sensors-22-07737-s001.zip › sensors-1924235-supplementary.pdf]

The Fabrication of Cesium Lead Bromide-Coated Cellulose Nanocomposites and Their Effect on the Detection of Nitrogen Gas

Bumjun Park [1,†], Haneul Kang [1,†], Soobin Han [1], Hyeong-U Kim [2], Youngjin Cho [3,*], Yun Suk Huh [1,*] and Sung-Min Kang [4,5,*]

[1] Department of Biological Sciences and Bioengineering, Inha University, 100 Inha-ro, Michuhol-gu, Incheon 22212, Korea
[2] Department of Plasma Engineering, Korea Institute of Machinery & Materials, Daejeon 34103, Korea
[3] Food Safety and Distribution Research Group, Korea Food Research Institute, 245 Nongsaengmyeong-ro, Iseo-myeon, Wanju-gun 55365, Jeollabuk-do, Korea
[4] Department of Green Chemical Engineering, Sangmyung University, 31 Sangmyungdae-gil, Cheonan 31066, Chungcheongnam-do, Korea
[5] Future Environment and Energy Research Institute, Sangmyung University, 31 Sangmyungdae-gil, Cheonan 31066, Chungcheongnam-do, Korea
* Correspondence: yjcho74@kfri.re.kr (Y.C.); yunsuk.huh@inha.ac.kr (Y.S.H.); smkang@smu.ac.kr (S.-M.K.)
† These authors contributed equally to this work.

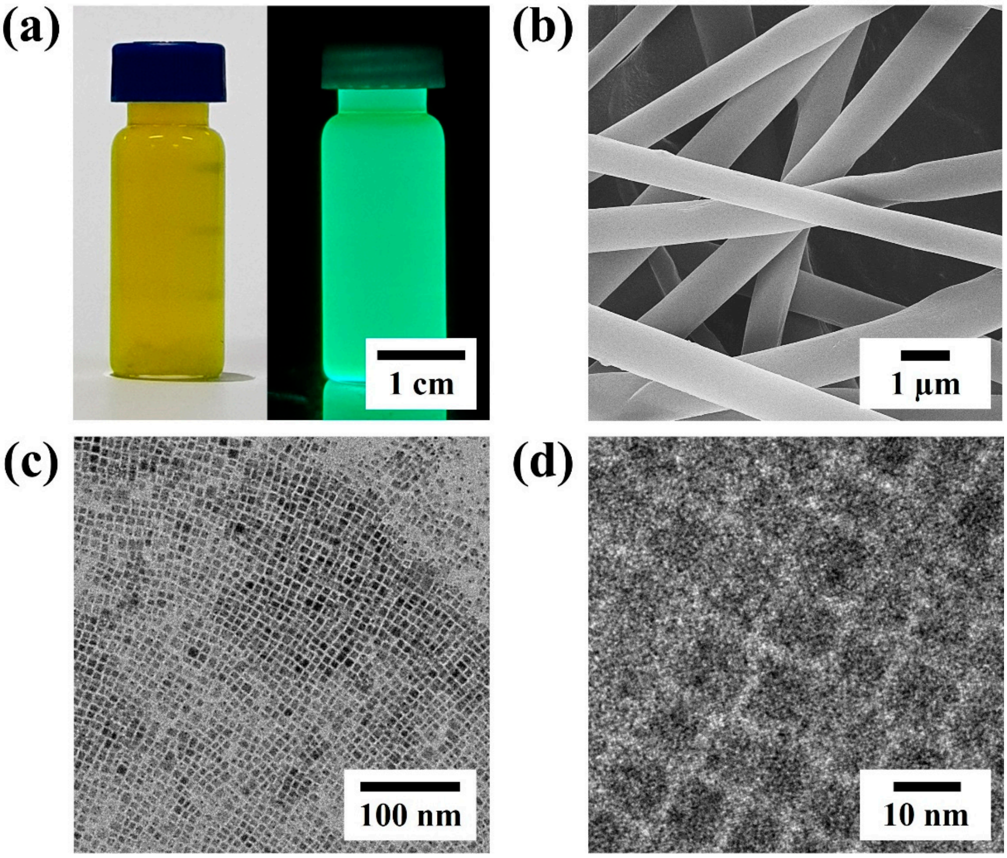

Figure S1. Morphological characterization; **(a)** $CsPbBr_3$ NCs under visible light and UV field, **(b)** scanning electron microscopy (SEM) image of CNFs, and transmission electron microscopy (TEM) image of $CsPbBr_3$ NCs at **(c)** low and **(d)** high magnification.

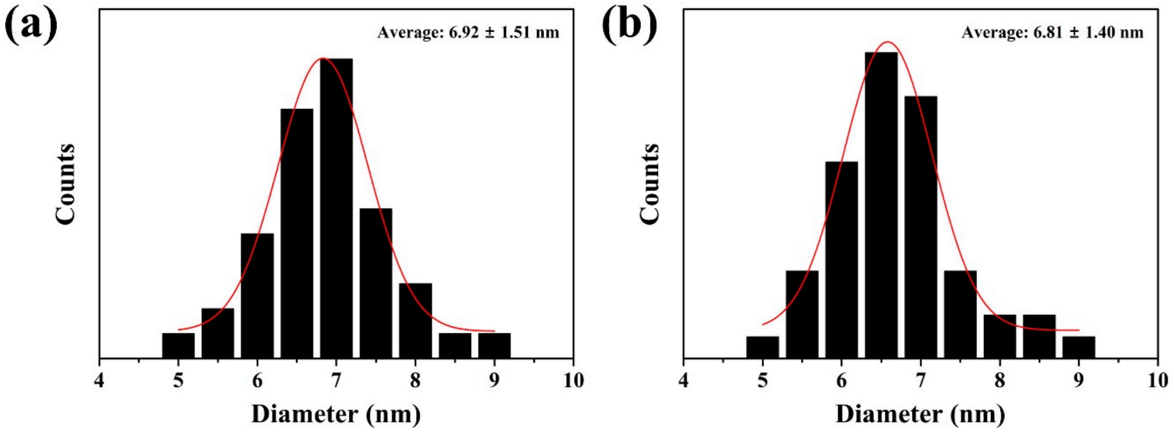

Figure S2. Size distribution graph of (a) $CsPbBr_3$ NCs and (b) NCs attached on the surface of CNFs.

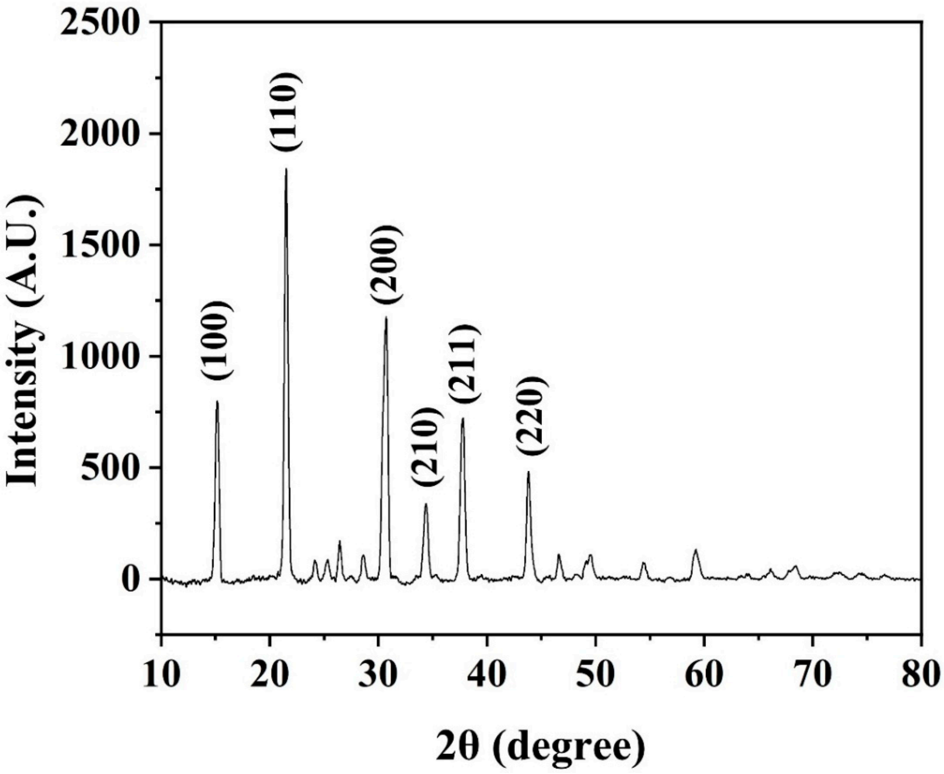

Figure S3. X-ray diffraction (XRD) pattern of fabricated $CsPbBr_3$ NCs through hot injection method.

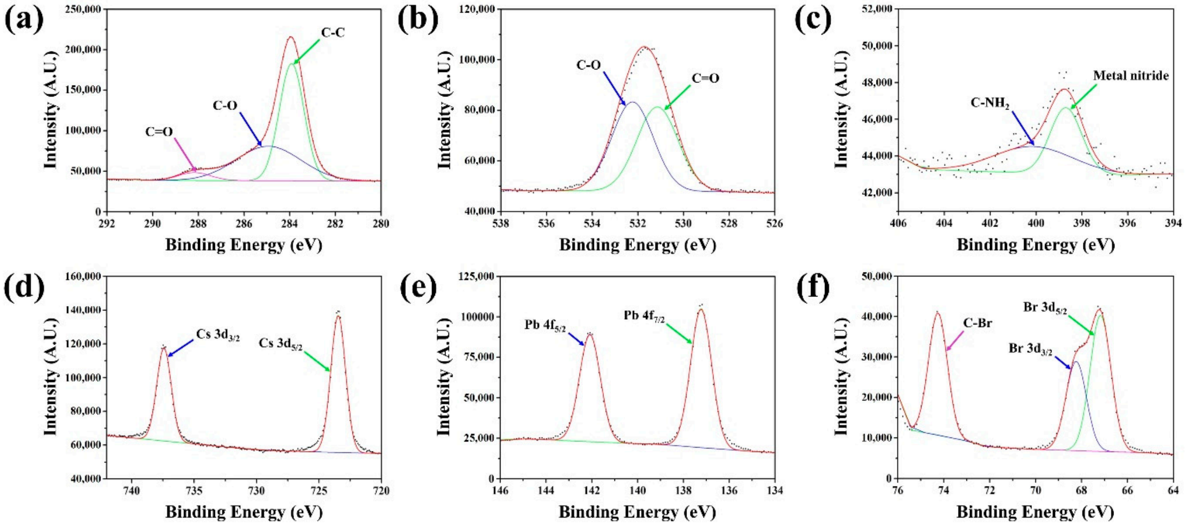

Figure S4. Narrow X-ray photoelectron spectroscopic (XPS) scan of (a) C 1s, (b) O 1s, (c) N 1s, (d) Cs 3d, (e) Pb 4f, and (f) Br 3d for $CsPbBr_3$ NFs.

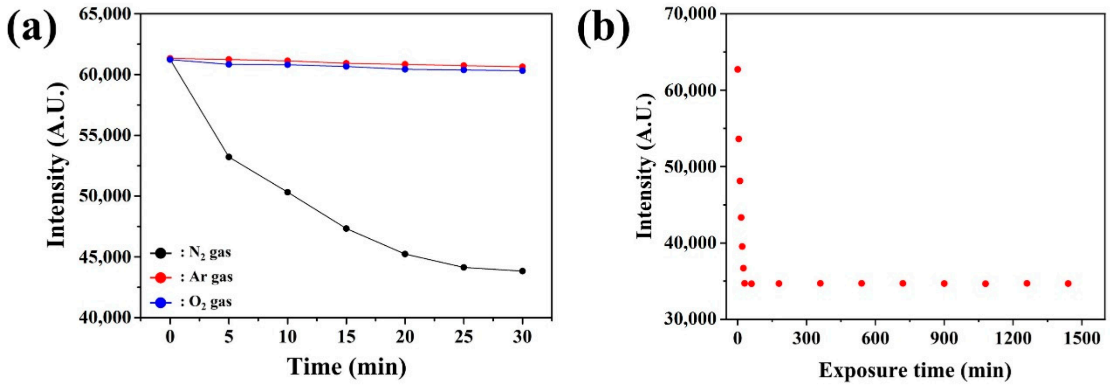

Figure S5. (a) Selectivity of fabricated gas sensor with various gas at a concentration of 10 ppm, and (b) repeatability analysis of fabricated gas sensors under atmosphere after detection of nitrogen gas.

Table S1. Deconvolution of the XPS spectra.

Elemental composition		Binding energy (eV)
C 1s	C–C	283.93
	C–O	285.45
	C=O	288.22
O 1s	C=O	531.12
	C–O	532.22
N 1s	Metal nitride	398.78
	C–NH_2	400.61
Cs 3d	Cs $3d_{5/2}$	723.43
	Cs $3d_{3/2}$	737.41
Pb 4f	Pb $4f_{7/2}$	137.22
	Pb $4f_{5/2}$	142.06
Br 3d	Br $3d_{5/2}$	67.19
	Br $3d_{3/2}$	68.10
	C–Br	74.24

Table S2. Comparison of gas sensors for detection of various types of nitrogen gas.

**Analytical technique**	**Detecting gas**	**LOD (μM)**	**Ref.**
Absorption spectra measurement	NH_3	58.72	[37]
Electrochemical measurement	NO	10.00	[38]
Amplified chemoresistor measurement	NO_2	0.22	[39]
$CsPbBr_3$ NFs-based photoluminescence measurement	N_2	28.57	This work
